# Impact of chronic hepatitis on cardiovascular events among type 2 diabetes patients in Taiwan pay-for-performance program

**DOI:** 10.1038/s41598-022-15827-x

**Published:** 2022-07-09

**Authors:** Yi-Jing Sheen, Chih-Cheng Hsu, Pei-Tseng Kung, Li-Ting Chiu, Wen-Chen Tsai

**Affiliations:** 1 Department of Health Services Administration, China Medical University, No. 100, Sec. 1, Jingmao Rd., Beitun Dist., Taichung, 406040 Taiwan; 2 Division of Endocrinology and Metabolism, Department of Internal Medicine, Taichung Veterans General Hospital, Taichung, Taiwan; 3 Department of Medicine, School of Medicine, National Yang Ming Chiao Tung University, Taipei, Taiwan; 4 Department of Public Health, China Medical University, Taichung, Taiwan; 5 Institute of Population Health Sciences, National Health Research Institutes, Zhunan, Taiwan; 6 Department of Healthcare Administration, Asia University, Taichung, Taiwan; 7 Department of Medical Research, China Medical University Hospital, China Medical University, Taichung, Taiwan

**Keywords:** Diseases, Endocrinology, Health care, Medical research

## Abstract

To investigate the impact of chronic hepatitis on cardiovascular events in patients with type 2 diabetes mellitus (T2DM). This nationwide retrospective cohort study included 152,709 adult patients (> 20 years) with T2DM enrolled in the National Health Insurance Diabetes Pay-for-Performance Program from 2008 to 2010 and followed up until the end of 2017. Patients were categorized into groups with hepatitis B, hepatitis C, fatty liver disease, and patients without chronic hepatitis. The incidence of cardiovascular events in patients with T2DM and hepatitis C (79.9/1000 person-years) was higher than that in patients with diabetes combined with other chronic hepatitis, or without chronic hepatitis. After adjusting for confounding factors, T2DM with fatty liver (adjusted hazard ratio [HR]: 1.10; 95% confidence interval [CI]: 1.07–1.13) and hepatitis C (adjusted HR: 1.09; 95% CI: 1.03–1.12) demonstrated a significantly higher risk of cardiovascular events. The adjusted visit-to-visit coefficient of variation of HbA1c and fasting blood glucose were associated with a high risk of cardiovascular events (HRs of the highest quartile were 1.05 and 1.12, respectively). Chronic hepatitis affects cardiovascular events in adult patients with T2DM. Glucose variability could be an independent risk factor for cardiovascular events in such patients.

## Introduction

Abnormal blood glucose metabolism is common in patients with chronic hepatitis, with approximately 96% of patients with liver cirrhosis developing insulin resistance and 30% developing diabetes^1^. According to a previous study, high levels of glutamic-pyruvic transaminase (GPT) can increase the risk of diabetes, and the effects are independent of obesity or high triglyceride levels^2^. Common types of chronic hepatitis that are thought to be related to blood glucose metabolism are hepatic insulin resistance, non-alcoholic fatty liver disease (NAFLD), chronic hepatitis B, and chronic hepatitis C^3^. Researchers in Taiwan have found that fasting blood glucose level is related to abnormal hepatic function. Increased fasting blood glucose and impaired insulin sensitivity were significantly associated with elevated hepatic function indicators such as gluconic transaminase and GPT^4^. A study in patients with hepatitis B also found that high fasting blood glucose was correlated with obesity and increased GPT^5^.

There is substantial evidence for the correlation of hepatitis C with diabetes. A Taiwanese study found that patients with hepatitis C had a relatively high risk of abnormal blood glucose, with levels as high as 67.9% even without tissue structural abnormalities such as liver fibrosis^6^. Hepatitis C with viremia is a risk factor for the development of type 2 diabetes mellitus (T2DM)^7^.

Fatty liver/NAFLD is known to be associated with an increased risk of diabetes. Insulin resistance in the liver is one of the etiologies of diabetes^8,9^. A Taiwanese study found that the prevalence of NAFLD in the population of persons with diabetes was 31.8%, whereas the prevalence of diabetes in the NAFLD population was 20.2%, which was significantly higher than that in the population without NAFLD (7.5%)^10^. A study on the relationship between chronic hepatitis and insulin resistance found that fasting blood glucose, C-peptide, and insulin resistance differed significantly from those in the control group even after adjusting for sex, age, and the etiology of liver cirrhosis^11^. Although both hepatitis B and C cause increased insulin resistance, NAFLD causes the highest resistance^12^. Notably, NAFLD and diabetes in Asian populations may not always be a manifestation of obesity. According to one study, 23.5% of patients with NAFLD were non-obese, and patients with NAFLD had significant metabolic abnormalities compared with non-NAFLD patients; however, metabolic abnormality indicators such as blood lipid levels were similar between obese and non-obese NAFLD patients^13^.

Correlations between chronic hepatitis and cardiovascular disease with diabetes also exist. The evidence for NAFLD and hepatitis C is relatively abundant. Lipids/inflammation/fibrosis of the liver may be common mechanisms but are important determinants of cardiovascular disease in patients with T2DM and NAFLD or hepatitis C^14,15^. NAFLD was previously correlated with increased cardiovascular disease in patients with diabetes^16^. NAFLD is a multi-system disease related to diabetes and cardiovascular disease, resulting in hepatocyte injury, liver cirrhosis, and liver cancer. Although it directly impacts the liver, death is mainly related to cardiovascular disease^17,18^. Whether in patients with or without diabetes, the correlation of hepatitis C with atherosclerosis and cardiovascular disease has been supported by considerable empirical evidence^19^. Extrahepatic effects of hepatitis C virus infection are not uncommon and are correlated with the incidence and mortality of cardiovascular disease^20^. As for the influence of hepatitis B infection on cardiovascular disease, the results remain controversial. A meta-analysis pointed out that there is no significant correlation between hepatitis B and coronary heart disease^21^.

The pay-for-performance program (P4P) of diabetes was designed as an integrated team-care program consists of doctors, nurses, pharmacists, and other medical workers, to provide thorough and comprehensive medical care^22,23^. In the P4P program, service fees for biochemistry examinations (such as fasting glucose level, HbA1c, renal function, lipid profiles, and proteinuria), fundus examinations, and diabetes self-management education are reimbursed, aimed mainly at monitoring chronic vascular complications of diabetes. Both T2DM and chronic hepatitis are prevalent globally; concurrent diseases may worsen some chronic complications and cause premature death. At present, the prevention and treatment of chronic complications of diabetes focus on large and small blood vessels, as well as integration and connection with chronic nephropathy, without active intervention regarding chronic hepatitis. Taiwan has been implementing the P4P program for several years; however, research and evidence regarding the impact of chronic hepatitis on subsequent complications of diabetes are lacking.

Therefore, we investigated the impact of chronic hepatitis on cardiovascular events to provide a reference for the development of comprehensive care strategies for preventable cardiovascular comorbidities among patients with T2DM.

## Results

In this study, 152,709 patients with T2DM (age 59.32 ± 11.67 years) who joined P4P from 2008 to 2010 were selected as the study population after screening (Table 1). The proportion of women (50.35%) was slightly higher than that of men; the follow-up period was at least 7 years (according to a 2017 report of the Ministry of Health and Welfare, the enrollment rate in the P4P until 2017 only accounted for 26.3–29.3% of the population^24^). Three common types of chronic hepatitis were observed in 17.83% of the study population: fatty liver (7.86%), hepatitis B (6.66%), and hepatitis C (3.31%). The highest proportion of patients was aged 55–64 years (32.1%). The types of chronic hepatitis according to age range were as follows: the prevalence of hepatitis B and fatty liver was the highest among patients aged 40–54 years, whereas the prevalence of hepatitis C was the highest among patients aged 55–64 years. Overall, patients with hepatitis B were the youngest (55.57 ± 10.54 years), and those with hepatitis C were the oldest (61.13 ± 10.01 years).

**Table 1:** The baseline characteristics of the incident cardiovascular disease in patients with T2DM.

Variables	Total		No liver-related disease		Hepatitis B		Hepatitis C		Fatty liver disease		p-value ^a^
	N	%	N	%	N	%	N	%	N	%	
Total	152,709	100.00	125,473	82.16	10,174	6.66	5056	3.31	12,006	7.86	–
**Sex**											< 0.001
Male	75,815	49.65	60,823	80.23	6328	8.35	2526	3.33	6138	8.10	
Female	76,894	50.35	64,650	84.08	3846	5.00	2530	3.29	5868	7.63	
**Duration of DM (years)**											< 0.001
≤ 1	23,150	15.16	18,372	79.36	1796	7.76	732	3.16	2250	9.72	
1–5	26,260	17.20	21,146	80.53	1867	7.11	891	3.39	2356	8.97	
5–10	22,014	14.42	17,910	81.36	1491	6.77	836	3.80	1777	8.07	
> 10	21,931	14.36	18,501	84.36	1120	5.11	806	3.68	1504	6.86	
Not available	59,354	38.87	49,544	83.47	3900	6.57	1791	3.02	4119	6.94	
**Age (years)**											< 0.001
20–39	7757	5.08	6042	77.89	711	9.17	104	1.34	900	11.60	
40–54	46,455	30.42	36,814	79.25	4176	8.99	1253	2.70	4212	9.07	
55–64	49,038	32.11	40,016	81.60	3298	6.73	1870	3.81	3854	7.86	
65–74	34,682	22.71	29,382	84.72	1646	4.75	1383	3.99	2271	6.55	
≥ 75	14,777	9.68	13,219	89.46	343	2.32	446	3.02	769	5.20	
Mean ± SD	59.32 ± 11.67		59.78 ± 11.74		55.57 ± 10.54		61.13 ± 10.01		57.02 ± 11.62		< 0.001
**Monthly salary (NTD)**											< 0.001
≤ 17,280	34,388	22.52	28,916	84.09	1963	5.71	960	2.79	2549	7.41	
17,281–22,800	58,322	38.19	47,629	81.67	3833	6.57	2439	4.18	4421	7.58	
22,801–36,300	28,527	18.68	23,397	82.02	1964	6.88	850	2.98	2316	8.12	
36,301–45,800	15,812	10.35	12,829	81.13	1194	7.55	461	2.92	1328	8.40	
≥ 45,801	15,660	10.25	12,702	81.11	1220	7.79	346	2.21	1392	8.89	
**CCI score**											< 0.001
0	97,806	64.05	83,871	85.75	4938	5.05	1800	1.84	7197	7.36	
1	33,761	22.11	25,667	76.03	3308	9.80	1754	5.20	3032	8.98	
2	12,938	8.47	10,112	78.16	964	7.45	738	5.70	1124	8.69	
≥ 3	8204	5.37	5823	70.98	964	11.75	764	9.31	653	7.96	
**DCSI score (mean ± SD)**	0.61 ± 0.95		0.62 ± 0.96		0.55 ± 0.89		0.68 ± 1.01		0.52 ± 0.85		< 0.001
0	93,835	61.45	76,560	81.59	6474	6.90	2969	3.16	7832	8.35	< 0.001
1	35,511	23.25	29,203	82.24	2386	6.72	1201	3.38	2721	7.66	
≥ 2	23,363	15.30	19,710	84.36	1314	5.62	886	3.79	1453	6.22	
**Hypertension**											< 0.001
No	66,137	43.31	53,468	80.84	5073	7.67	2123	3.21	5473	8.28	
Yes	86,572	56.69	72,005	83.17	5101	5.89	2933	3.39	6533	7.55	
**Gout**											< 0.001
No	141,581	92.71	116,454	82.25	9476	6.69	4664	3.29	10,987	7.76	
Yes	11,128	7.29	9019	81.05	698	6.27	392	3.52	1019	9.16	
**BMI (kg/m**^**2**^**)**^**b**^											< 0.001
BMI < 18.5	1159	0.76	987	85.16	79	6.82	52	4.49	41	3.54	
18.5 ≤ BMI < 24	47,270	30.95	39,303	83.15	3241	6.86	1737	3.67	2989	6.32	
24 ≤ BMI < 27	24,825	16.26	20,297	81.76	1665	6.71	837	3.37	2026	8.16	
BMI ≥ 27	42,984	28.15	34,403	80.04	3042	7.08	1228	2.86	4311	10.03	
Not performed	36,471	23.88	30,483	83.58	2147	5.89	1202	3.30	2639	7.24	
**Smoking**											< 0.001
Never	144,528	94.64	118,966	82.31	9532	6.60	4712	3.26	11,318	7.83	
Ever	8181	5.36	6507	79.54	642	7.85	344	4.20	688	8.41	
**Alcohol use**											< 0.001
Never	149,014	97.58	122,573	82.26	9876	6.63	4939	3.31	11,626	7.80	
Ever	3695	2.42	2900	78.48	298	8.06	117	3.17	380	10.28	
**Hospital level**											< 0.001
Medical center	29,057	19.03	23,788	81.87	1773	6.10	847	2.91	2649	9.12	
Regional	54,780	35.87	45,199	82.51	3500	6.39	1963	3.58	4118	7.52	
District	25,893	16.96	20,877	80.63	1791	6.92	945	3.65	2280	8.81	
Clinic	42,979	28.14	35,609	82.85	3110	7.24	1301	3.03	2959	6.88	
**HbA1c (%)**											
1st time (Mean ± SD)	7.92 ± 1.68		7.93 ± 1.68		7.83 ± 1.68		7.82 ± 1.71		7.87 ± 1.66		< 0.001
< 7	48,911	32.03	39,567	80.90	3545	7.25	1778	3.64	4021	8.22	< 0.001
7–9	72,271	47.33	59,740	82.66	4635	6.41	2273	3.15	5623	7.78	
> 9	31,527	20.65	26,166	83.00	1994	6.32	1005	3.19	2362	7.49	
Average of 1st year (mean ± SD)	7.71 ± 1.30		7.72 ± 1.30		7.64 ± 1.27		7.63 ± 1.31		7.66 ± 1.27		< 0.001
CV (× 100)^c^	8.68		8.60		8.99		9.84		8.82		< 0.001
SD (× 100)	69.46		68.96		71.21		77.27		69.93		< 0.001
Adjusted CV (× 100)^c^	7.97		7.90		8.25		9.03		8.10		< 0.001
CV ≤ 4.28	38,221	25.03	32,005	83.74	2399	6.28	961	2.51	2856	7.47	< 0.001
4.28 < CV ≤ 6.60	38,083	24.94	31,507	82.73	2458	6.45	1121	2.94	2997	7.87	
6.60 < CV ≤ 10.12	38,256	25.05	31,284	81.78	2589	6.77	1318	3.45	3065	8.01	
CV > 10.12	38,149	24.98	30,677	80.41	2728	7.15	1656	4.34	3088	8.09	
**Fasting blood glucose (mg/dl)**											
1st time (mean ± SD)	151.03 ± 52.55		151.32 ± 52.49		149.66 ± 52.20		151.30 ± 58.01		149.03 ± 50.91		< 0.001
< 80	3086	2.02	2521	81.69	210	6.80	143	4.63	212	6.87	< 0.001
80–130	56,544	37.03	46,036	81.42	3935	6.96	1890	3.34	4683	8.28	
> 130	93,079	60.95	76,916	82.64	6029	6.48	3023	3.25	7111	7.64	
Average of 1st year (mean ± SD)	148.69 ± 38.26		148.76 ± 38.18		148.16 ± 38.55		149.67 ± 40.39		147.99 ± 37.88		0.020
CV (× 100)^c^	19.55		19.51		19.78		21.58		18.91		< 0.001
SD (× 100)	3028.09		3020.67		3069.56		3374.86		2924.43		< 0.001
Adjusted CV (× 100)^c^	17.78		17.75		17.96		19.61		17.22		< 0.001
CV ≤ 9.91	39,533	25.89	32,597	82.46	2615	6.61	1119	2.83	3202	8.10	< 0.001
9.91 < CV ≤ 15.23	37,699	24.69	31,036	82.33	2520	6.68	1094	2.90	3049	8.09	
15.23 < CV ≤ 22.95	37,739	24.71	30,882	81.83	2531	6.71	1278	3.39	3048	8.08	
CV > 22.95	37,738	24.71	30,958	82.03	2508	6.65	1565	4.15	2707	7.17	
**LDL-C (mg/dl) (mean ± SD)**^**b**^	109.32 ± 32.63		109.80 ± 32.66		106.94 ± 31.60		101.90 ± 30.88		109.38 ± 33.46		< 0.001
< 100	61,315	40.15	49,685	81.03	4329	7.06	2546	4.15	4755	7.76	< 0.001
100–130	54,498	35.69	44,844	82.29	3680	6.75	1666	3.06	4308	7.90	
> 130	36,896	24.16	30,944	83.87	2165	5.87	844	2.29	2943	7.98	
**Creatinine (mg/dl) (mean ± SD)**	1.00 ± 0.57		1.01 ± 0.59		1.00 ± 0.56		1.04 ± 0.62		0.94 ± 0.40		< 0.001
eGFR (ml/min/1.73 m^2^) ≥ 90	36,759	24.07	29,539	80.36	2777	7.55	999	2.72	3444	9.37	< 0.001
60 ≤ eGFR ≤ 89	77,939	51.04	63,369	81.31	5549	7.12	2667	3.42	6354	8.15	
45 ≤ eGFR ≤ 59	24,178	15.83	20,499	84.78	1277	5.28	839	3.47	1563	6.46	
30 ≤ eGFR ≤ 44	9911	6.49	8645	87.23	380	3.83	380	3.83	506	5.11	
15 ≤ eGFR ≤ 29	3130	2.05	2746	87.73	139	4.44	134	4.28	111	3.55	
eGFR < 15	792	0.52	675	85.23	52	6.57	37	4.67	28	3.54	
**SGPT (u/l) (Mean ± SD)**^**b**^	31.43 ± 31.34		27.85 ± 24.65		46.46 ± 53.03		63.81 ± 62.87		39.77 ± 30.94		< 0.001
< 35	37,501	24.56	32,676	87.13	1775	4.73	748	1.99	2302	6.14	< 0.001
35–100	12,275	8.04	8360	68.11	1386	11.29	887	7.23	1642	13.38	
> 100	1284	0.84	549	42.76	222	17.29	334	26.01	179	13.94	
Not performed	101,649	66.56	83,888	82.53	6791	6.68	3087	3.04	7883	7.76	
**Triglycerides (mg/dl) (mean ± SD)**	152.34 ± 157.07		153.59 ± 152.79		139.55 ± 163.05		124.13 ± 103.61		162.02 ± 205.40		< 0.001
< 150	95,912	62.81	77,858	81.18	7093	7.40	3785	3.95	7176	7.48	< 0.001
150–200	25,271	16.55	21,096	83.48	1437	5.69	648	2.56	2090	8.27	
> 200	31,526	20.64	26,519	84.12	1644	5.21	623	1.98	2740	8.69	
**UACR (mg/dl)**											< 0.001
< 30	99,672	65.27	81,365	81.63	7076	7.10	3066	3.08	8165	8.19	
30–300	27,809	18.21	22,950	82.53	1650	5.93	1052	3.78	2157	7.76	
> 300	8286	5.43	7011	84.61	440	5.31	372	4.49	463	5.59	
Not performed	16,942	11.09	14,147	83.50	1008	5.95	566	3.34	1221	7.21	

^a^χ^2^ test, ANOVA.

^b^*T2DM* type 2 diabetes mellitus, *BMI* body mass index, *LDL-C* low density lipoprotein, *eGFR* estimated glomerular filtration rate, *SGPT* serum glutamic-pyruvic transaminase, *UACR* urine albumin-to-creatinine ratio (by random urine specimen).

^c^CV = SD/Mean; adjusted CV = CV/√(n/n − 1).

As for the recorded durations of diabetes, regardless of the status of chronic hepatitis, the highest proportion of patients had diabetes for 1–5 years. More than 60% of the patients (64.05%) had a Charlson comorbidity index of 0. Similarly, 61.45% of the patients had a score of 0 on the DCSI. Among all the patients, 56.7% had hypertension, 28.15% of the patients with body mass index (BMI) values were obese (BMI ≥ 27), and 5.36% had a history of smoking. The average HbA1c level was 7.92 ± 1.68% at the time of enrollment and reduced to 7.71 ± 1.30% in the first year after enrollment. The adjusted CV% of HbA1c of the entire population was 7.97%, with the highest value (9.03%) in the hepatitis C group and a value of 7.90% in the non-chronic hepatitis group. The average pre-meal blood glucose level was 151.03 ± 52.55 mg/dL at the time of enrollment and reduced to 148.69 ± 38.26 mg/dL in the first year after enrollment. The adjusted CV% of the pre-meal blood glucose level of the entire population was 17.78%, with the highest value (19.61%) in the hepatitis C group and the lowest value (17.22%) in the fatty liver group. About 40% of the patients had a low-density lipoprotein level of < 100 mg/dL at the time of enrollment, 75% of the patients had an estimated glomerular filtration rate of ≥ 60 mL/min/1.73 m^2^ at the time of enrollment, and 65% of the patients had a urine albumin-to-creatinine ratio < 30 mg/g.

Table 2 presents the incidence rate per 1000 person-years of cardiovascular disease in T2DM patients with chronic hepatitis. The incidence rate of cardiovascular disease was the highest (79.91/1000 person-years) in patients with diabetes and hepatitis C, followed by patients with fatty liver (66.76/1000 person-years), hepatitis B, and without chronic hepatitis. There was no significant difference between patients with diabetes and hepatitis B or without chronic hepatitis.

**Table 2:** The incident rate of cardiovascular disease in patients with T2DM.

Variables	Total		Cardiovascular disease				p-value^a^	Incident rate (per 1000 person-year)	p-value^b^
			No		Yes				
	N	%	N	%	N	%			
Total	152,709	100.00	87,732	57.45	64,977	42.55		66.37	
**Patients with/without chronic hepatitis**							< 0.001		
No liver-related disease	125,473	82.16	71,983	57.37	53,490	42.63		66.50	–
Hepatitis B	10,174	6.66	6292	61.84	3882	38.16		58.33	< 0.001
Hepatitis C	5056	3.31	2675	52.91	2381	47.09		79.91	< 0.001
Fatty liver disease	12,006	7.86	6782	56.49	5224	43.51		66.76	0.788
**Sex**							< 0.001		
Male	75,815	49.65	44,746	59.02	31,069	40.98		63.60	–
Female	76,894	50.35	42,986	55.90	33,908	44.10		69.13	< 0.001
**Duration of DM (years)**							< 0.001		
≤ 1	23,150	15.16	15,860	68.51	7290	31.49		45.56	–
1–5	26,260	17.20	16,562	63.07	9698	36.93		53.96	< 0.001
5 − 10	22,014	14.42	12,729	57.82	9285	42.18		64.28	< 0.001
> 10	21,931	14.36	11,223	51.17	10,708	48.83		81.04	< 0.001
Not performed	59,354	38.87	31,358	52.83	27,996	47.17		77.19	< 0.001
**Age (year)**							< 0.001		
20–39	7757	5.08	6097	78.60	1660	21.40		27.44	–
40–54	46,455	30.42	31,499	67.81	14,956	32.19		44.71	< 0.001
55–64	49,038	32.11	28,121	57.35	20,917	42.65		65.32	< 0.001
65–74	34,682	22.71	16,147	46.56	18,535	53.44		94.64	< 0.001
≥ 75	14,777	9.68	5868	39.71	8909	60.29		131.08	< 0.001
Mean ± SD	59.32 ± 11.67		57.10 ± 11.59		62.33 ± 11.10				
**Monthly salary (NTD)**							< 0.001		
≤ 17,280	34,388	22.52	18,548	53.94	15,840	46.06		75.96	–
17,281–22,800	58,322	38.19	32,782	56.21	25,540	43.79		69.33	< 0.001
22,801–36,300	28,527	18.68	17,250	60.47	11,277	39.53		58.97	< 0.001
36,301–45,800	15,812	10.35	9613	60.80	6199	39.20		58.49	< 0.001
≥ 45,801	15,660	10.25	9539	60.91	6121	39.09		58.38	< 0.001
**CCI score**							< 0.001		
0	97,806	64.05	59,990	61.34	37,816	38.66		56.70	–
1	33,761	22.11	17,709	52.45	16,052	47.55		78.66	< 0.001
2	12,938	8.47	6231	48.16	6707	51.84		93.50	< 0.001
≥ 3	8204	5.37	3802	46.34	4402	53.66		121.50	< 0.001
**DCSI score (mean ± SD)**	0.61 ± 0.95		0.51 ± 0.84		0.75 ± 1.07				
0	93,835	61.45	57,598	61.38	36,237	38.62	< 0.001	57.66	–
1	35,511	23.25	19,597	55.19	15,914	44.81		70.61	< 0.001
≥ 2	23,363	15.30	10,537	45.10	12,826	54.90		102.47	< 0.001
**Hypertension**							< 0.001		
No	66,137	43.31	45,531	68.84	20,606	31.16		43.13	–
Yes	86,572	56.69	42,201	48.75	44,371	51.25		88.53	< 0.001
**Gout**							< 0.001		
No	141,581	92.71	82,159	58.03	59,422	41.97		65.04	–
Yes	11,128	7.29	5573	50.08	5555	49.92		84.89	< 0.001
**BMI (kg/m**^**2**^**)**^**c**^							< 0.001		
BMI < 18.5	1159	0.76	766	66.09	393	33.91		54.21	0.060
18.5 ≤ BMI < 24	47,270	30.95	28,624	60.55	18,646	39.45		59.67	–
24 ≤ BMI < 27	24,825	16.26	14,263	57.45	10,562	42.55		65.07	< 0.001
BMI ≥ 27	42,984	28.15	23,631	54.98	19,353	45.02		70.02	< 0.001
Not performed	36,471	23.88	20,448	56.07	16,023	43.93		72.64	< 0.001
**Smoking**							< 0.001		
Never	144,528	94.64	82,683	57.21	61,845	42.79		66.93	–
Had	8181	5.36	5049	61.72	3132	38.28		56.98	< 0.001
**Alcohol use**							< 0.001		
Never	149,014	97.58	85,428	57.33	63,586	42.67		66.68	–
Had	3695	2.42	2304	62.35	1391	37.65		54.64	< 0.001
**Hospital level**							< 0.001		
Medical center	29,057	19.03	18,717	64.41	10,340	35.59		51.97	–
Regional	54,780	35.87	32,178	58.74	22,602	41.26		63.44	< 0.001
District	25,893	16.96	14,271	55.12	11,622	44.88		72.42	< 0.001
Clinic	42,979	28.14	22,566	52.50	20,413	47.50		77.54	< 0.001
**HbA1c (%)**									
1st time (mean ± SD)	7.92 ± 1.68		7.87 ± 1.65		7.98 ± 1.71				
< 7	48,911	32.03	28,944	59.18	19,967	40.82	< 0.001	63.17	–
7–9	72,271	47.33	41,498	57.42	30,773	42.58		65.95	< 0.001
> 9	31,527	20.65	17,290	54.84	14,237	45.16		72.54	< 0.001
Average of 1st year (mean ± SD)	7.71 ± 1.30		7.64 ± 1.25		7.80 ± 1.35				
CV (× 100)^d^	8.68		8.57		8.83				
SD (× 100)	69.46		67.89		71.57				
Adjusted CV (× 100)^d^	7.97		7.87		8.11				
CV ≤ 4.28	38,221	25.03	23,101	60.44	15,120	39.56	< 0.001	59.04	–
4.28 < CV ≤ 6.60	38,083	24.94	22,004	57.78	16,079	42.22		64.91	< 0.001
6.60 < CV ≤ 10.12	38,256	25.05	21,486	56.16	16,770	43.84		69.24	< 0.001
CV > 10.12	38,149	24.98	21,141	55.42	17,008	44.58		73.01	< 0.001
**Fasting blood glucose (mg/dl)**									
1st time (mean ± SD)	151.03 ± 52.55		149.42 ± 50.47		153.20 ± 55.15				
< 80	3086	2.02	1673	54.21	1413	45.79	< 0.001	77.46	–
80–130	56,544	37.03	33,144	58.62	23,400	41.38		64.19	< 0.001
> 130	93,079	60.95	52,915	56.85	40,164	43.15		67.37	< 0.001
Average of 1st year (mean ± SD)	148.69 ± 38.26		147.17 ± 36.57		150.74 ± 40.34				
CV (× 100)^d^	19.55		18.84		20.51				
SD (× 100)	3028.09		2885.59		3220.20				
Adjusted CV (× 100)^d^	17.78		17.14		18.65				
CV ≤ 9.91	39,533	25.89	24,349	61.59	15,184	38.41	< 0.001	57.16	–
9.91 < CV ≤ 15.23	37,699	24.69	22,322	59.21	15,377	40.79		61.66	< 0.001
15.23 < CV ≤ 22.95	37,739	24.71	21,259	56.33	16,480	43.67		68.56	< 0.001
CV > 22.95	37,738	24.71	19,802	52.47	17,936	47.53		80.22	< 0.001
**LDL-C (mg/dl) (mean ± SD)**^**c**^	109.32 ± 32.63		108.85 ± 31.93		109.94 ± 33.55				
< 100	61,315	40.15	35,405	57.74	25,910	42.26	< 0.001	66.24	–
100–130	54,498	35.69	31,623	58.03	22,875	41.97		64.87	0.021
> 130	36,896	24.16	20,704	56.11	16,192	43.89		68.83	< 0.001
**Creatinine (mg/dl) (mean ± SD)**	1.00 ± 0.57		0.95 ± 0.48		1.07 ± 0.67				
eGFR(ml/min/1.73 m^2^) ≥ 90	36,759	24.07	24,458	66.54	12,301	33.46	< 0.001	47.94	–
60 ≤ eGFR ≤ 89	77,939	51.04	46,281	59.38	31,658	40.62		61.36	< 0.001
45 ≤ eGFR ≤ 59	24,178	15.83	11,975	49.53	12,203	50.47		86.00	< 0.001
30 ≤ eGFR ≤ 44	9911	6.49	3846	38.81	6065	61.19		123.98	< 0.001
15 ≤ eGFR ≤ 29	3130	2.05	927	29.62	2203	70.38		174.18	< 0.001
eGFR < 15	792	0.52	245	30.93	547	69.07		186.07	< 0.001
**SGPT(u/l) (mean ± SD)**^**c**^	31.43 ± 31.34		32.39 ± 31.78		30.15 ± 30.70				
< 35	37,501	24.56	20,958	55.89	16,543	44.11	< 0.001	68.38	–
35 − 100	12,275	8.04	7337	59.77	4938	40.23		60.86	< 0.001
> 100	1,284	0.84	820	63.86	464	36.14		55.62	< 0.001
Not performed	101,649	66.56	58,617	57.67	43,032	42.33		66.45	0.002
**Triglycerides (mg/dl) (mean ± SD)**	152.34 ± 157.07		147.78 ± 150.26		158.50 ± 165.61				
< 150	95,912	62.81	56,942	59.37	38,970	40.63	< 0.001	62.23	–
150–200	25,271	16.55	13,897	54.99	11,374	45.01		71.86	< 0.001
> 200	31,526	20.64	16,893	53.58	14,633	46.42		75.23	< 0.001
**UACR (mg/dl)**							< 0.001		
< 30	99,672	65.27	60,398	60.60	39,274	39.40		58.83	–
30–300	27,809	18.21	14,645	52.66	13,164	47.34		77.14	< 0.001
> 300	8286	5.43	3492	42.14	4794	57.86		106.50	< 0.001
Not performed	16,942	11.09	9197	54.29	7745	45.71		80.93	< 0.001

^a^Log-rank test.

^b^Univariate Poisson regression.

^c^*T2DM* type 2 diabetes mellitus, *BMI* body mass index, *LDL-C* low density lipoprotein, *eGFR* estimated glomerular filtration rate, *SGPT* serum glutamic-pyruvic transaminase, *UACR* urine albumin-to-creatinine ratio (by random urine specimen).

^d^CV = SD/Mean; adjusted CV = CV/√(n/n − 1).

Table 3 presents the Cox proportional hazard model indicating the relative risk of cardiovascular disease in T2DM patients with and without chronic hepatitis. After adjusting for confounding factors, patients with diabetes and fatty liver had the highest risk of cardiovascular disease (adjusted hazard ratio [HR]: 1.10; 95% confidence interval [CI]: 1.07–1.13), followed by those with hepatitis C (adjusted HR: 1.09; 95% CI: 1.03–1.12), hepatitis B, and those without chronic hepatitis. There was no significant difference between patients with diabetes and hepatitis B or without chronic hepatitis.

**Table 3:**
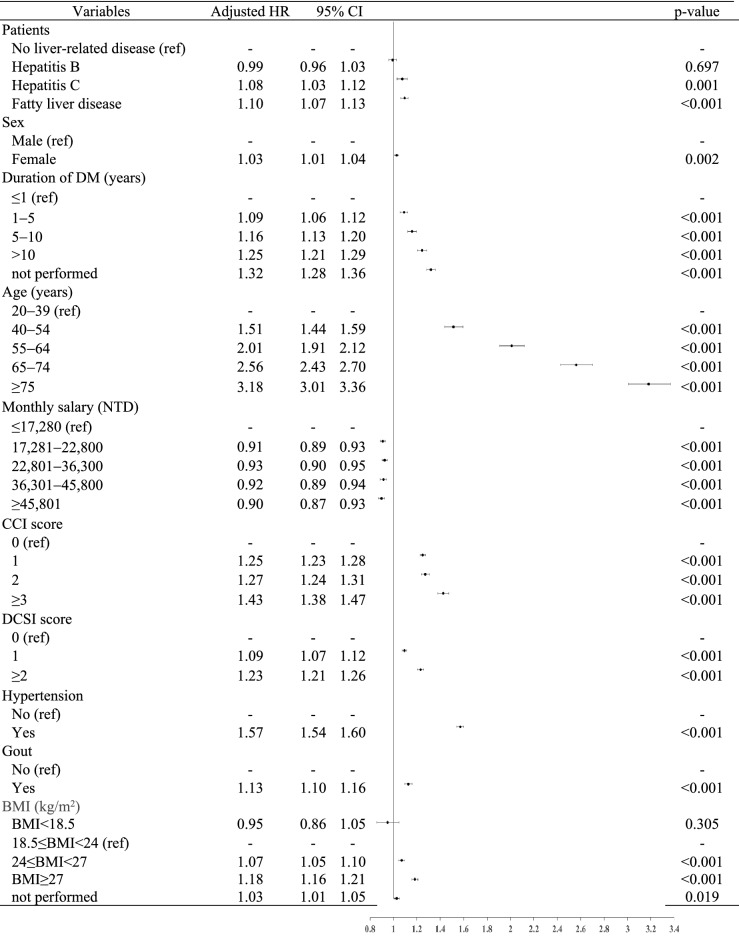

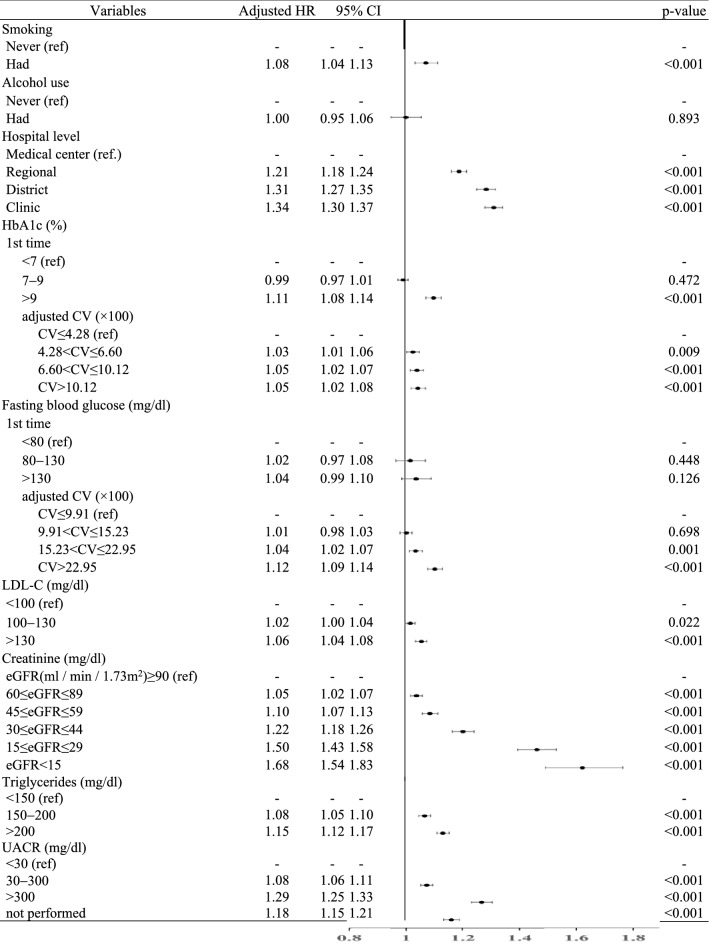
The risk and influencing factors of incident cardiovascular disease in patients with T2DM.

*T2DM* type 2 diabetes mellitus, *CCI* Charlson comorbidity index, *DCSI* diabetes complications severity index, *BMI* body mass index, *LDL-C *low density lipoprotein, *eGFR* estimated glomerular filtration rate, *UACR* urine albumin-to-creatinine ratio (by random urine specimen), *HR* hazard ratio.

As for other variables, slightly more women (adjusted HR: 1.03; 95% CI: 1.01–1.04) developed cardiovascular disease. A higher risk of cardiovascular disease was associated with older age (adjusted HR of patients aged > 75 years: 3.18; 95% CI: 3.01–3.36), a longer disease duration, more complications, heavier bodyweight, (adjusted HR of patients with BMI > 27: 1.18; 95% CI: 1.16–1.21), hypertension, gout, and/or smoking history. Furthermore, patients with HbA1c > 9%, a higher visit-to-visit CV% of pre-meal blood glucose/HbA1c, worsening renal function (adjusted HR of patients with chronic kidney disease stage 5 and estimated glomerular filtration < 15: 1.68; 95% CI: 1.54–1.83), low-density lipoprotein > 130 mg/dL (adjusted HR: 1.06; 95% CI: 1.04–1.08), higher triglyceride levels, and severe proteinuria (measured by urine albumin-to-creatinine ratio of random urine specimen, UACR) also had a higher risk of cardiovascular disease (Table 3).

## Discussion

After adjusting for confounding factors, we found that compared with T2DM patients without hepatitis, those with fatty liver had the highest risk of cardiovascular disease. The correlation between diabetes and liver diseases is universal across races and regions. A significant association between chronic hepatitis and insulin resistance was found even after adjusting for sex, age, and the etiology of chronic liver diseases^11^. Hepatitis C had a higher risk of increased blood glucose, and viremia is an independent risk factor for developing T2DM^7^. Both hepatitis B, C, and fatty liver increase insulin resistance; however, NAFLD have the most significant influence^12^.

This again confirmed the findings of several previous studies on the mechanisms and epidemiology of cardiovascular disease. NAFLD was correlated with increased cardiovascular and cancer mortality in patients with diabetes^16^. Steatosis and chronic inflammation of the liver progressing to fibrosis and abnormal metabolism are important determinants of cardiovascular disease as complications of T2DM^21,22^. NAFLD is correlated with metabolic syndrome and cardiovascular and cerebrovascular diseases^25^. Some researchers state that NAFLD with diabetes metabolic abnormality and atherosclerosis are like two sides of the same coin^26^.

Hepatitis C correlates with atherosclerosis and cardiovascular disease. The use of antiviral drugs for treating hepatitis C can reduce the risk of dyslipidemia and cardiovascular disease in patients with diabetes, hepatitis C^27^, and carotid atherosclerosis^28^, as well as reduce low-density lipoprotein and intima thickness in patients with diabetes and hepatitis C^29^. The treatment of chronic hepatitis is effective not only for the liver itself but also for correcting metabolic abnormalities and managing complications of diabetes caused by chronic hepatitis, which may be reversed and improved through the treatment of viral hepatitis. Therefore, early-stage screening and intervention for chronic hepatitis have significance for patients with diabetes.

There was a slightly higher risk of cardiovascular events among women in this study (adjusted HR: 1.01), which is not consistent with previous studies. Male sex has been identified as a risk factor for cardiovascular disease, irrespective of diabetes status^30,31^. This result is attributed to the protective effect of estrogen against cardiovascular disease. However, this protective effect declines with age in women because of menopause. Some studies have disagreed with this sentiment; maintaining that lifestyle and socio-economic pressure are the main factors^31,32^. Epidemiological research in Taiwan on causes of death in patients with T2DM found that the rate of female patients with diabetes who died because of cardiovascular disease in 2014 was 12.53%, which was slightly higher than that of male patients in the same year (12.00%)^33^. Therefore, cardiovascular disease in female patients with diabetes also poses an issue that should not be ignored.

The results of the analysis were consistent with those of previous studies on the traditional risk factors of cardiovascular disease. The older the patient, the longer the disease duration, the more complications, and the heavier the body weight (BMI > 27), the higher the risk of cardiovascular disease. Hypertension, gout, smoking history, poor renal function, and low-density lipoprotein > 130 mg/dL were also associated with a higher risk of cardiovascular disease.

Regarding abnormal blood glucose metabolism, patients with HbA1c > 9% and a higher visit-to-visit adjusted CV% of pre-meal blood glucose/HbA1c had a higher risk of cardiovascular disease. The correlation of blood glucose variation with cardiovascular disease and other complications of diabetes has received considerable attention recently. With improvements in blood glucose monitoring technology, research has found that aside from episodes of hyperglycemia and hypoglycemia and poor long-term control as reflected by high HbA1c, blood glucose variation is an important factor leading to disease deterioration and complications^34^. Variations in pre-meal blood glucose are significantly correlated with mortality from cardiovascular disease^35^. Furthermore, visit-to-visit variation of HbA1c is also correlated with macrovascular complications of newly diagnosed diabetes^36^. In the future, visit-to-visit variation of pre-meal blood glucose/HbA1c should receive more attention in blood glucose control among patients with diabetes. Reducing variations in blood glucose is an important goal in the treatment and prevention of high-risk cardiovascular complications.

### Study limitations

This study has some limitations. First, National Health Insurance Research Database and ICD diagnosis codes were used to define relevant diseases. Thus, a relatively strict diagnostic definition was adopted (patients who had been hospitalized once or had more than three outpatient visits within 365 days and who were diagnosed with diabetes or chronic hepatitis with ICD diagnosis codes were included); however, the definition had been validated in a previous study with a sensitivity of 96.9% and a positive predictive value of 93.9%^37^ to avoid coding bias. Additionally, the medications of the patients were not analyzed, since it is a confounding factor extremely challenging to control. Each study participant may receive inconsistent prescriptions in the follow-up period, including drug type, drug dose, drug adherence, and medication duration. Therefore, we analyzed clinical data to evaluate the conditions of traditional risk control. We adjusted the biochemistry exam, including LDL-C, and triglycerides to evaluate the situation of treatment to targets, instead of the complicated situation of medication use; however, the pleiotropic effects of medications were not evaluated. As a disadvantage of all retrospective studies, some clinical data were not available and could not be analyzed. Since this is a nationwide study, the laboratory methods of each hospital are not available; however, the principles of quality assurance, quality control, and quality management should be confirmed while the hospitals upload their data to the Data Science Center of the National Health Insurance Administration. Notably, this study focused on adult patients with T2DM in Taiwan who joined P4P. The contents of the P4P include regular follow-up laboratory tests, clinical examinations, and health education, which could explain the involvement of a relatively young population. Moreover, male patients might be less available for follow-up visits due to work restrictions, explaining why we observed a slightly higher number of women in the study population^38^.

### Study strengths

The strengths of the study were that unlike most previous nationwide studies, clinical data were available in the National Health Insurance Research Database, and our study combined large and representative nationwide databases with laboratory data in Taiwan.

In conclusion, chronic hepatitis has varying effects on cardiovascular events in adult patients with T2DM. The variability of fasting blood glucose and HbA1c are independent risk factors for cardiovascular events in patients with T2DM. Patients with diabetes and fatty liver or hepatitis C should be educated on the risk factors for cardiovascular disease actively and as early as possible. In addition to controlling the traditional risk factors, such as blood glucose and blood lipid control, weight loss, smoking cessation, and reduction of glucose variation are also important goals.

## Methods

### Research subjects

**Figure 1:**
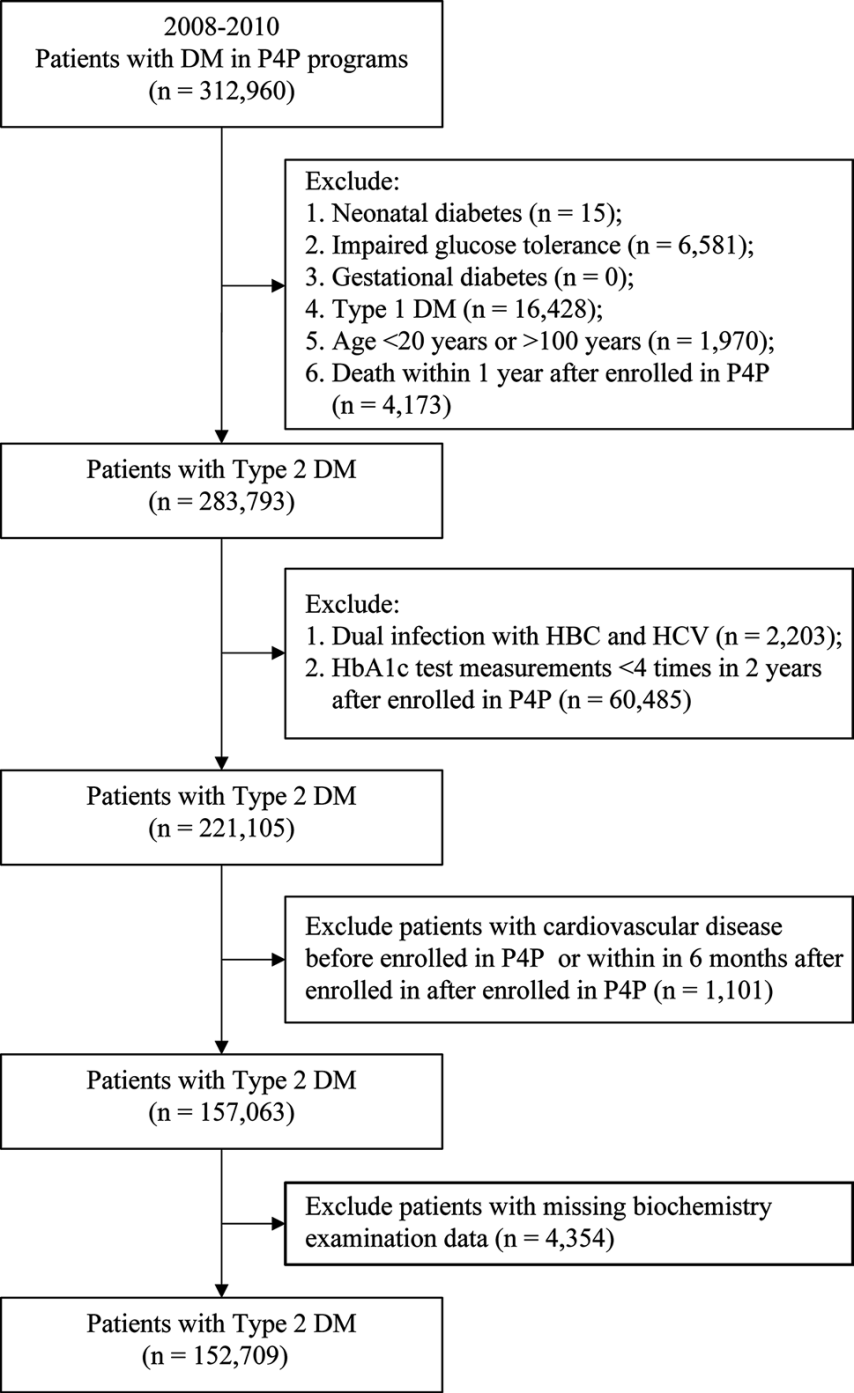
Flowchart for study subject selection. *DM* diabetes mellitus, *P4P* pay-for-performance, *HBV* hepatitis B virus, *HCV* hepatitis C virus.

Patients with T2DM who joined the P4P from 2008 to 2010 were enrolled. Patients with a confirmed diagnosis of T2DM were defined as those who were hospitalized at least once or came in for outpatient visits at least three times within 1 year and had a primary or secondary diagnosis International Classification of Diseases (ICD) code “250,” “250.00,” or “250.02”^38,39^. Among them, patients with type 1 DM “250.x1” * or “250.x3;” gestational DM “648.0” or “648.8;” neonatal DM “775.1;” abnormal glucose tolerance test “790.2;” age < 20 years or > 100 years; and those who died within 1 year of joining P4P were excluded. Finally, 283,793 patients were included (Fig. 1). Based on the status of comorbid chronic hepatitis at enrollment, the patients were divided into four groups: no comorbid chronic hepatitis, named as “No chronic hepatitis”; comorbid liver B, named as “Hepatitis B” group; comorbid liver, named as “Hepatitis C” group; patients without viral hepatitis and with comorbid fatty liver were named as the “Fatty liver disease” group and were followed-up until the end of 2017. The “no comorbid chronic hepatitis” group was used as the reference group to analyze the correlation between different types of chronic hepatitis and the risk of cardiovascular disease.

### Ethics statements

The National Health Insurance Research Database (NHIRD) is derived from Taiwan’s mandatory National Health Insurance program was established by the National Health Insurance Administration Ministry of Health and Welfare and maintained by the National Health Research Institute (NHRI). The patient identifications in the National Health Insurance Research Database have been scrambled and de-identified by the Taiwan government, and the database is commonly used for different types of research such as in medical, and public health fields. Thus, informed consent was waived by the Research Ethics Committee of the China Medical University, and the study protocol was approved by the research ethics committee of China Medical University and Hospital (IRB number: CMUH106-REC3-153) and was conducted in accordance with the principles of the Declaration of Helsinki.

### Data sources

This retrospective cohort study analyzed data from the National Health Insurance Research Database of the “Applied Health Research Data Integration Service from National Health Insurance Administration”. The data included outpatient prescriptions and treatments, outpatient prescriptions and medical orders, inpatient medical expense lists, inpatient medical expense and medical order lists, insurance details of persons, major injury and illness, medical institution master files, diagnosis, and P4P education records.

### Definitions of variables

Hepatitis B: Those with ICD-9 070.2, 070.20, 070.21, 070.22, 070.23, 070.3070.31, 070.32, or 070.33 or ICD-10 B16, B17.0, B18.0, B18.1, or B19.1 as the primary and secondary diagnosis during two outpatient visits or one hospitalization within 365 days of study enrollment.

Hepatitis C: Those with ICD-9 070.41, 070.44, 070.51, or V02.62 or ICD-10 B17.10, B17.11, B18.2, B19.20, B19.21, or Z22.52 as the primary and secondary diagnosis during two outpatient visits or one hospitalization within 365 days of study enrollment.

NAFLD: Those with ICD-9 571.8, 571.9, or ICD-10 K74.4, K74.5, K74.60, K74.69, K76.0, K76.9, etc. as the primary and secondary diagnosis during two outpatient visits or one hospitalization within 365 days of study enrollment, and without the occurrence of a hepatitis B or C code, for whom the first hospital visit within 365 days was defined as the date of diagnosis. Patients with concurrent viral hepatitis and NAFLD were classified as having viral hepatitis.

Age-based categorization included 20–39, 40–54, 55–64, 65–74, and ≥ 75 years age groups. Monthly salary was divided into five grades, namely ≤ NTD 17,280, NTD 17,281–22,800, NTD 22,801–36,300, NTD 36,301–45,800, and ≥ NTD 45,801. Charlson comorbidity index was divided into 0, 1, 2, and ≥ 3 after excluding scores correlated with independent or dependent variables^40^.

The diabetes complications severity index (DCSI) was scored as 0, 1, and ≥ 2 points. The DCSI was calculated based on the classification and scoring method proposed by Young et al. If the patient had no complication, the score would be 0; for each complication, 1 point would be added; if the complication was serious, 2 points would be added. Based on this calculation method, the maximum score was 13 points^41^.

Cardiovascular disease: Those with ICD-9 398.91, 402.xx, 404.xx, 410.xx–414.xx, 422.xx, 425.xx or 428.xx, or ICD-10 I09.81, I11, I13, I20–I22, I24, I25, I40–I43, I50, R09.89, etc. as the primary and secondary diagnosis during two outpatient visits or one hospitalization within 365 days of study enrollment^42^.

Calculation of the coefficient of variation (CV% = standard deviation/mean) of HbA1c and fasting blood glucose: All measurements in the first year were used, and if the measurements were taken less than four times in the first year, measurements taken up to the second year were included. If measurements were taken less than four times in the 2 years, the patient would be excluded.

Adjusted CV = CV/√ (n/n − 1): When the examination data were limited, the examination times would affect the result of the CV. In this case, a relatively correct result of the CV with a reduced effect of the examination times could be obtained by correcting the examination times.

### Analytical methods

Descriptive and inferential statistics were carried out according to the research objectives and framework. All research tests were based on a significance level of α = 0.05, and all statistical analyses were conducted using SAS software for Windows, version 9.4 (SAS Institute Inc., Cary, NC, USA). Descriptive statistics such as frequency, percentage, average, and standard deviation were used to describe the dependent and independent variables to be investigated in this study. This study adopted descriptive statistics to present the demographic characteristics, status of comorbidities, blood biochemical indicators, health status, economic factors, and medical care provider characteristics of patients with diabetes. The incidence of cardiovascular disease in patients with T2DM with chronic hepatitis per 1000 person-years was tested using univariate Poisson regression. The relative risks of cardiovascular disease in the four groups were calculated using a Cox proportional hazards model.

## Data Availability

Data are available from the Data Science Center of the National Health Insurance Administration (NHIA), the Ministry of Health and Welfare (MOHW) (https://www.mohw.gov.tw/mp-2.html), Taiwan. All interested researchers can apply for using the database managed by the NHIA. Due to legal restrictions imposed by the Taiwanese government related to the Personal Information Protection Act, the database cannot be made publicly available. Any raw data are not allowed to be brought out from the Data Science Center. The restrictions prohibited the authors from making the minimal data set publicly available.
